# Energy-Efficient Clustering Multi-Hop Routing Protocol in a UWSN

**DOI:** 10.3390/s21020627

**Published:** 2021-01-18

**Authors:** Nhat-Tien Nguyen, Thien T. T. Le, Huy-Hung Nguyen, Miroslav Voznak

**Affiliations:** 1 Department of Telecommunications, VSB-Technical University of Ostrava, 708 00 Ostrava, Czech Republic; tien.nn@sgu.edu.vn; 2 Faculty of Electronics and Telecommunications, Sai Gon University, Ho Chi Minh City 700000, Vietnam; thien.lett@sgu.edu.vn (T.T.T.L.); nghhung@sgu.edu.vn (H.-H.N.)

**Keywords:** underwater wireless sensor network, energy-efficient, clustering, depth-based routing

## Abstract

Underwater wireless sensor networks are currently seeing broad research in various applications for human benefits. Large numbers of sensor nodes are being deployed in rivers and oceans to monitor the underwater environment. In the paper, we propose an energy-efficient clustering multi-hop routing protocol (EECMR) which can balance the energy consumption of these nodes and increase their network lifetime. The network area is divided into layers with regard to the depth level. The data sensed by the nodes are transmitted to a sink via a multi-hop routing path. The cluster head is selected according to the depth of the node and its residual energy. To transmit data from the node to the sink, the cluster head aggregates the data packet of all cluster members and then forwards them to the upper layer of the sink node. The simulation results show that EECMR is effective in terms of network lifetime and the nodes’ energy consumption.

## 1. Introduction

The ecosystem on Earth mainly consists of water, which covers more than 75% of its surface. Water covers the Earth in many forms as rivers, lakes, and oceans and plays a crucial role in human life and for other animals. Technological developments have allowed sensors to be deployed for the exploration of nature’s forest, river, and lake environments. These sensors are embedded with smart sensing and intelligent computing and are capable of communicating with each other. Underwater wireless sensor networks (UWSNs) consist of many autonomous sensor nodes, which are considered homogeneous nodes and limited in energy [1,2]. These underwater sensor nodes are deployed in rivers or seas to detect the characteristics present in the water environment, such as temperature, current flow, pressure, and water quality. This type of data is aggregated at a data processing station according to different types of applications. From observation of the environment, humans gain many benefits for many applications, such as environmental monitoring, disaster forecasting, military surveillance, and assisted navigation. In particular, detailed examination of the underwater environment assists humans in the observation of marine life, disaster forecasting, water pollution monitoring, and sea exploration [3,4]. The hardware architecture of underwater sensor nodes is described in [5]. The internal architecture of an underwater sensor node consists of several modules: memory, an acoustic modem, a sensor, sensor interface circuitry, a control processor unit (onboard controller), and a power supply. The underwater sensor node can be used to measure underwater characteristics such as temperature, density, acidity, chemicals, conductivity, pH, hydrogen, dissolved methane gas, and turbidity.

**Figure 1:**
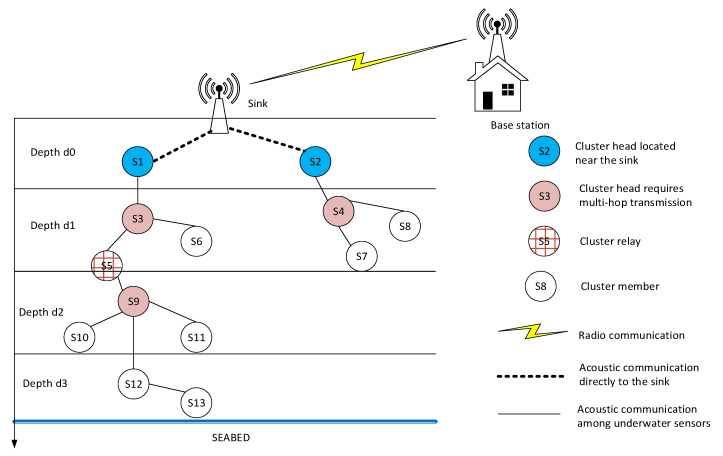
Underwater wireless sensor network (UWSN) model.

Figure 1 depicts the network model of UWSNs [1,2,3]. The UWSN consists of underwater sensor nodes, a sink node, and an onshore base station. In Figure 1, the nodes located near the sink directly transmit data to the sink, while the other nodes form clusters. The underwater sensor nodes transmit their data to the sink at the surface. The sink then forwards the aggregated data to the nearest base station on the mainland. The sink node is equipped with two types of transceivers: (1) a radio transceiver, which can communicate with the base station by radio frequency; and (2) an acoustic transceiver for communication with the sensor nodes. The underwater node is embedded with an acoustic transceiver for transmission between nodes. The transmission medium of the underwater environment is different to that of a terrestrial wireless sensor network (TWSN) with respect to channel modeling, path loss, and topology [2,3]. The underwater wireless sensor nodes are moved by currents with velocities of around 1–3 m/s. The acoustic channel is modeled differently from the radio propagation channel.

In both TWSNs and UWSNs, data packets are sent from a sensor node to the base station via either a one-hop path or a multi-hop path. It is necessary to route the data packet from one node to another node without loss. Since each sensor node is equipped with a battery, which may be difficult to replace or charge, the re-transmission of data packets may consume high energy, which results in a short network lifetime or many dead nodes. In TWSNs, many clustering protocols have been developed to minimize the energy consumption of sensors while successfully transmitting data packets to the destination. In TWSNs, all nodes are homogeneous and energy-constrained, and a low-energy adaptive clustering hierarchy (LEACH) successfully reduces the energy consumption of nodes while maintaining their network lifetime [6]. In [7], a clustering protocol for UWSNs was reviewed, demonstrating that the clustering protocol is suitable for underwater transmission. As with clustering in TWSNs, the clustering protocol is designed to reduce the control information, resulting in a reduction in the overall energy consumption, extended node lifetime, and greater network reliability. However, as a result of the effect of the current on the underwater sensor node, the cluster protocol should maintain connectivity to the network to ensure full coverage. In [8], a depth-based routing protocol (DBR) was developed to route a packet from any sensor to the sink node according to the node’s depth level. The DBR allows nodes to forward data to the node at a higher layer near the sink.

In the present paper, we develop a clustering protocol with regard to the location of nodes and residual energy of nodes. We propose an energy-efficient clustering multi-hop routing protocol (EECMR) for UWSNs so that each node can maintain its transmission link to the sink node at the surface and its neighbor sensor nodes. Our contribution through this work is as follows:

- The underwater sensor nodes update the routing path and cluster to connect to the sink without disruption. The sink collects data, which are then transmitted to an onshore gateway.
- Three types of node: cluster head (CH), cluster member (CM), and cluster relay (CR). If a node is the cluster head, the role of the node can be an aggregation node. If a node is the cluster relay, the node forwards data from two clusters at two depth levels. Otherwise, nodes are designated as a cluster member and only send data to the cluster head.
- The underwater sensors calculate the weight according to the node’s depth and residual energy to elect to become the cluster head.
- The routing protocol takes into account the number of cluster members and load balancing parameters so that the node selects the best route to the destination.

The remainder of the paper is organized as follows: Section 2 reviews the existing clustering routing protocols for UWSNs; Section 3 introduces and analyzes the energy-efficient clustering multi-hop routing protocol; Section 4 presents an evaluation of network performance in comparison to the LEACH protocol and DBR routing protocol; Section 5 concludes the paper.

## 2. Related Works

UWSNs have attracted many researchers over the last decade because of their enormous variety of application. Many literature reviews of routing protocols for UWSNs categorizing the routing protocols in terms of the routing strategy have been presented [9]. The routing protocol for UWSNs has two types: localization-based and localization-free protocols. The localization-based routing protocol requires the location of nodes to find the routing path from the node to the sink. Despite this, the localization-free protocol uses the depth measurement of the sensor node to establish the routes to the sink. The depth of the sensor node is measured by the pressure sensors equipped on the node. The localization-free protocol has more advantages than the other in terms of scalability. Due to the mobility of nodes, the routes can be constructed by using the depth if the network topology changes. 

In [7], several cluster protocols for UWSNs are reviewed in terms of cluster stability, delay efficiency, load balancing, and energy efficiency. The network requires a capability of 100% coverage due to the long transmission range and high node mobility. The clustering protocols in UWSNs select the cluster heads and cluster members depending on the location of nodes, types of generated packets, priority of nodes, the energy consumption, and the latency of data. A clustering protocol should achieve high network performance with respect to a high packet delivery ratio, low latency, and a long network lifetime. Some clustering protocols are reviewed in detail in this section. 

In [10], a clustered-based routing protocol allows the sensors to form clusters according to their depth and energy. The localization-free routing protocol, which is called the energy-efficient routing protocol based on layers and unequal clusters (EERBLC), selects the routing path according to the link quality and residual energy. It presumes that each sensor node does not need its location to set up a cluster. A node calculates a cost according to its residual energy and forwarding ratio. In this protocol, the header nodes selection procedure does not consider the duration of the cluster head and the location of nodes according to the sink. If nodes play the role of the cluster head for a long duration of time, it may become a hot spot, causing a dead node. 

Another study applied LEACH in UWSNs to establish a cluster [11]. The study also improved LEACH, providing Controlled-LEACH (C-LEACH). C-LEACH deploys a control node at the center of a network’s topology, and the control nodes process the clustering protocol, resulting in a longer network lifetime compared to LEACH. The C-LEACH protocol may take a long time to process the cluster algorithm because the control nodes should transmit to all nodes in the network. 

In [12], the authors developed an adaptive node clustering routing protocol for smart ocean UWSNs based on an optimization technique. The network is divided into a cluster in which the cluster head is selected according to the available energy in the sensor node. The re-transmission may cause high latency or duplicated packets at the receiver while selecting the cluster members. 

In [13], network deployment is divided into layers in which each layer has a fixed depth value and area width. The multi-layer cluster-based energy-efficient (MLCEE) routing protocol increases network lifetime while reducing the end-to-end delay of a packet. The MLCEE protocol allows the node to calculate its layer; the node then commences broadcasting messages to form a cluster. Since the network is divided into many depth layers, the node adopts the role of forwarder depending on its residual energy and probability.

In [14], a topology control energy balance protocol (TCEB) was implemented for UWSNs. The protocol considers the node’s energy and path loss as the factors to select a cluster head. The topology control protocol is represented as a non-cooperative game. Each node can become the cluster head depending on the set of strategies and set of pay-offs for the game. The pay-off function is denoted as the function of residual energy and the path loss of one-hop distance and aims to balance the energy consumption of all nodes. 

In [15], another clustering protocol scheme was applied for multi-hop routing in UWSNs. The scheme allows nodes to change energy consumption while maintaining excellent network performance. The energy optimization clustering algorithm (EOCA) requires a node to communicate to neighbors within the effective communication range. Each node calculates the total transmission delay between the node and the sink and then elects to become a cluster head according to the value of the total transmission delay. The depth of each node was also used to find a forwarder before sending the packet to the sink. The nodes send data packets to the cluster head or directly to the sink; therefore, the energy consumption at the nodes can be balanced. 

In [16], an energy-balanced unequal layering clustering (EULC) algorithm was designed for UWSNs to reduce energy consumption in inter-cluster communications. The EULC creates clusters of varying size within the same layer to solve the ‘‘hot spot’’ issue. The depth of network deployment is divided into layers based on the communication radius. The sensor nodes calculate the weight with respect to the residual energy of the node, the distance to the sink node, and the node degree. The node with the highest weight elects to become the cluster head. The node stores the information of the neighbor cluster head and then calculates the routing selected function to all the neighbor cluster heads according to the distance between the node and the residual energy. The node selects the next-hop cluster head with the smallest selected routing function value.

In [17], the author implemented a simplified balanced energy adaptive routing (S-BEAR) based on a dynamic cluster K-means algorithm. The cluster heads are selected randomly. The nodes join the cluster with the minimum Euclid distance to the cluster head. The cluster head then transmits data to the sink using multi-hop communications.

In [18], autonomous unmanned vehicles (AUVs) were deployed underwater for cluster formation, cluster head selection, and scheduling the transmission and wake-up sleep cycle. Since the cluster heads have to wait for the AUVs to collect data, packet delay may increase as a result of the waiting time at the cluster head. 

In [19], a clustering vector-based forwarding algorithm (CVBF) performed clustering with the assistance of a virtual sink for each cluster. The network space is divided into equal cuboids, each cuboid being considered a cluster in which the node near the sink adopts the role of virtual sink. 

In [20], the authors presented an energy-optimized path unaware layered routing protocol (E-PULRP) for UWSNs. The network is divided into layers, forming a layered structure around the central sink node. A potential relay node is identified from each layer. The transmission around the sink is divided into a set of concentric shells which produce the layered structure. Communication from the node to the sink follows a multi-hop path through the relay node at each layer. Therefore, the E-PULRP protocol preserves energy consumption. 

There remains some issues and challenges of clustering protocols in UWSNs. The type of nodes and the depth of nodes should be considered because the nodes may generate different types of data such as temperature, density, acidity, chemicals, conductivity, pH, and hydrogen which are categorized into different types of priority level. Therefore, nodes which generate high traffic priority should have higher priority and higher reliability to transmit data to the sink. In addition, the nodes located near the sink always aggregate data from other nodes which may cause a hot spot and dead nodes. Therefore, nodes should change the cluster head role after some time. 

In [21], the authors reviewed and compared the routing protocols to select the forwarding node in a UWSN. Most of the routing protocols are multi-hop localization-free protocols which are energy-efficient and reliable. Therefore, the routing protocols for UWSNs should consider the depth of the node, energy consumption, and multi-hop routes in order to develop an efficient, reliable routing protocol. In addition, the clustering protocol for UWSNs shows the benefits in terms of high network coverage, high connectivity, and adaptivity to the dynamic environment. It should be noted that the existing routing protocols have some limitations. However, it is necessary to consider the role of the cluster relay and cluster head duration in minimizing energy consumption. The longer the nodes are at the cluster head, the more energy the nodes consume. In the paper, we propose a clustering protocol which takes into account the residual energy and the depth of nodes. The proposed protocol is described in the following section. A qualitative comparison of the clustering protocols is presented and discussed in Section 3.

## 3. Energy-Efficient Clustering Multi-Hop Routing Protocol

### 3.1. Network Model

The network scenario consists of *N* dynamic nodes which are randomly and sparsely deployed in a three-dimensional scenario *L × L × L*. Figure 1 illustrates the network. The data source is the sensed data in the water medium. The data are collected by the underwater sensor nodes. The sensed data are temperature, current flow, and pressure [1,2,3,4]. Underwater sensor nodes are equipped with acoustic modems to communicate with other nodes in the water medium. The sink node is deployed at the center of the surface and equipped with both acoustic and radio frequency (RF) modems; the sink’s acoustic modems receive data from the underwater sensor nodes, and the RF modem transmits data to the base station on the shore. We assumed that our network scenario was similar to the networks in [8,9,10]. The underwater sensor nodes are mobile due to water currents with velocities of around 1–3 m/s; therefore, the topology changes rapidly. A table of notations is listed in Table 1. The assumptions of the network can be described as follows:

- The node knows its location and the location of the sink node upon first deployment.
- Nodes can become either the cluster head, cluster relay, or cluster member.
- The cluster head is rotated between the sensor nodes to conserve energy.

The depth of deployment can be divided into layers, where each layer is defined by the transmission range of the node or sink, as given in (1):

(1) $$n_{depth}=\frac{L}{Tx},$$

where *L* is the depth of network deployment and *Tx* is the transmission range of the node or sink. 

In the paper, we divide the depth of the network, as shown in Figure 1. The depth of the network is divided into four cases: (1) the sink node at the surface, denoted *D*_0_; (2) nodes stay near the surface, whose depth is *D*_1_; (3) nodes stay in the water, whose depth is *D*_2_; (4) nodes stay near the seabed, whose depth is *D*_3_, where *D*_3_ > *D*_2_ > *D*_1_ > *D*_0_. The role of the nodes can be explained as follows:

- Each layer has several clusters, each cluster having one cluster head and a cluster member. For example, node *S*_3_ is the cluster head at depth *D*_1_ and node *S*_9_ is the cluster head at depth *D*_2_.
- Nodes located at the border of two layers can adopt the role of cluster relay, which forwards the data of the deeper layer to the sink. For example, node *S*_5_ becomes a cluster relay which forwards the data of cluster *S*_9_ to cluster *S*_3_ and then transmits to the sink node.
- Node *S*_13_ located at the seabed takes the multi-hop routing path to the sink via clusters of *S*_12_–*S*_9_–*S*_5_–*S*_3_–*S*_1_–sink. The propagation loss model of the underwater acoustic channel is assumed, as in [3]. Since the signals propagate vertically, attenuation increases, which is proportional to increasing distance. The transmission loss is calculated as follows:

(2) $$TL=k_{TL}\times 10\mathrm{lg}r+\alpha_{TL}r\times 10^{-3},$$

where *TL* represents the transmission loss in dB; *k_TL_* is the spreading factor, which indicates the spreading loss, its value depending on the water depth and corresponding to the propagation geometry; *k_TL_* = 1 in shallow water and cylindrical spreading; *k_TL_* = 2 for deep water and spherical spreading; α*_TL_* represents the absorption coefficient of the medium, which depends on the frequency, its unit being dB/km; *r* is the distance between the receiver and transmitter. The absorption coefficient for the medium is measured in dB/km for *f* in kHz; the equation for calculation is as follows:

(3) $$\alpha_{TL}(f)=0.11\frac{f^{2}}{1+f^{2}}+44\frac{f^{2}}{4100+f^{2}}+2.75\times 10^{-4}f^{2}+0.003,$$

The absorption coefficient increases with increasing frequency. In UWSNs, the frequency is approximately 30 kHz; the absorption coefficient is less than 10 dB/km. The absorption coefficient is derived for chemical absorption in seawater in terms of acoustic frequency, pressure, acidity, temperature, and salinity. The constant values are calculated using Thorp’s expression, which denotes the relaxation frequency for different chemical absorptions, the value of the acidic component and pH of the seawater, and the depth pressure of the seawater. Interested readers can refer to [22] for more details. 

**Figure 2:**
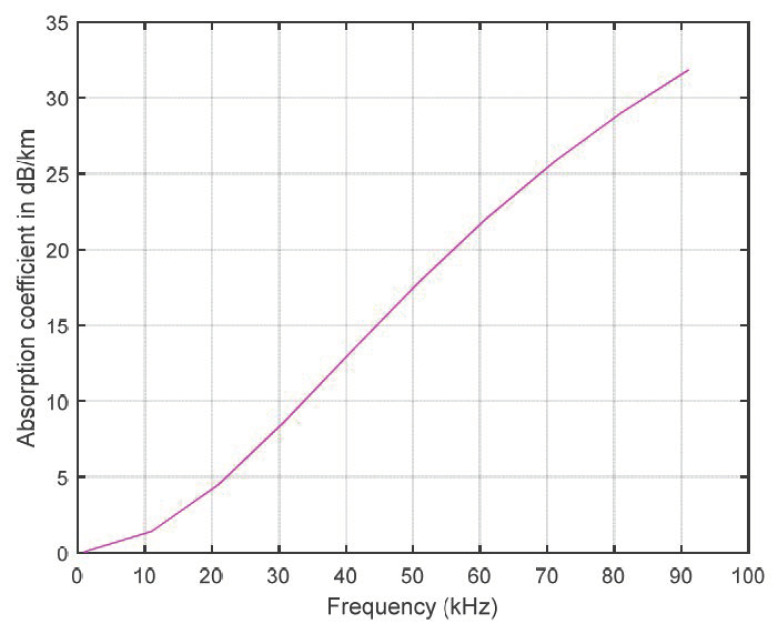
Absorption coefficient for the medium.

Based on the energy consumption model presented in [3,8,9], the least transmission power required at the transmitter to achieve power level *P*_0_ at the receiver can be expressed by

(4) $$P_{tx}=P_{0}\times d^{2}\times 10^{\left(\alpha_{TL}(f)/10\right)},$$

where *P*_0_ is the received power, *d* is the distance between the transmitter and receiver, and α*_TL_*(*f*) is the absorption coefficient, which is shown in Figure 2.

Since the characteristic of an acoustic wave in an underwater transmission medium is different from that of a radio wave, the energy consumption of wireless sensor networks cannot be applied to UWSNs. In the present paper, we apply the energy consumption model of the underwater acoustic channel as adopted in [3,12]. To transmit *k* bits of data over distance *d* with a data rate *R*, the energy consumed is defined as follows:

(5) $$E_{Tx}(k,d)=k\times E_{elec}+\frac{k}{R}P_{tx},$$

where *E_elec_* is the energy consumption to route 1 bit of data, and *P_Tx_* is the transmitted power, which is shown in equation (4).

To receive *k* bits of data, the receiver radio energy consumption can be expressed as

(6) $$E_{Rx}(k)=kP_{r},$$

where *P_r_* is a constant dependent on the device.

To fuse *k* bits of data, the energy consumption can be defined as

(7) $$E_{DA}(k)=k\times E_{DA0}$$

where *E_DA_*_0_ is the energy consumed by fusing one bit of data, which can be taken as 5 nJ/bit.

Since nodes are mobile due to the water current, we deploy random movement for nodes during the operating time. The current velocity is 1–3 m/s.

### 3.2. Energy-Efficient Clustering Multi-Hop Routing Protocol

The proposed energy-efficient clustering multi-hop routing protocol (EECMR) consists of two phases: a set-up phase and a steady-state phase. Cluster formation consists of sub-phases: broadcast information, cluster head selection, cluster formation, and scheduling transmission. 

In EECMR, the node becomes a CH, CM, or CR. For example, the node becomes a CH in the first round, the node becomes a CR in the second round, and the node may become a CM in the third round. Each node calculates a weight value based on the residual energy and distance to the sink. The weight of each node is used to decide whether or not to become a cluster head for the current round. The weight of *S_i_* is calculated as follows:

(8) $$W_{i}=\frac{\Delta t}{T}+\alpha \frac{d(i,S_{0})}{L}+\beta \frac{E_{Res}}{E_{Init}}$$

where *d*(*i*,*S*_0_) denotes the distance between node *S_i_* and the sink, *E_Res_* denotes the residual energy, *E_Init_* denotes the initial energy, α and β denote the coefficient such that α + β = 1, Δ*t* denotes the time for which the nodes adopt the role of cluster head, and *T* is the total operating time.

The EECMR protocol is described in Algorithm 1. At the start *t*_0_, the node does not have information about the surrounding environment. In State 1 from lines 3 to 7, each node broadcasts a “*HELLO*” message to the neighbors. The message includes information of the location and estimated distance to the sink. According to the successfully received “*HELLO*” message, nodes store their neighbors’ information in order to select the cluster, which is shown in lines 8 to 11. To prevent a long waiting time, the timeout value is set to the maximum propagation delay of one hop, as in [8]. The maximal propagation delay is calculated as τ = *Tx*/*v*_0_, where *Tx* is the maximum transmission range and *v*_0_ = 1.5 × 10^3^ m/s is the speed of sound propagation in the water.
**Algorithm 1. Energy-Efficient Clustering Multi-Hop Routing Protocol.****Input:** node ID, position, initial energy, generated packets **Output:** cluster {cluster ID, CH, CM}, CRsState 1: Network Initialization**For** each node *S_i_*  Calculate weight *W_i_* as in (8)  Broadcast *HELLO* = {node ID, position, *D_m_*, *W_i_*, residual energy, history of cluster head}**End For****While***t* < *timeout* do  Receive *HELLO* from other nodes  Create list of neighbors at each node *Nei*(*S_i_*) = {*Nei*(*S_i_*) ∪ *S_j_* | *d*(*i*,*j*) < *R*}**End While**State 2: Nodes elect to become cluster head**For** each node *S_i_*  {*Sk*, *Max*(*W_k_*)} = max{(*W_i_*,*W_j_*), *S_j_* ϵ *Nei*(*S_i_*)}  **If** *S_i_* has the *Max*(*W_k_*)    Broadcast *JOIN* = {Cluster ID, maximum number of cluster member, *D_m_*}    Elect to become cluster head  **Else**    Wait for *JOIN* message from the cluster head  **End If****End For****State 3. Relay node selection****For** All nodes are not assigned as CH  **If** *S_j_* receives *JOIN* messages from two nodes at *D_m_* and *D_m_*_−1_, respectively    Broadcast *RELAY* = {node ID, *D_m_*, Cluster ID(*D_m_*), Cluster ID(*D_m_*_−1_)}    Become a relay node which can coordinate the routing path  **Else**    Send the *RESPONSE* message to the CH    *RESPONSE* = {node ID, cluster ID, *D_m_*}  **End If****End For**State 4. Multi-hop routing path creation**For** all nodes that receive RELAY message  Find *D_m_* and node ID *S_k_* of RELAY message  **If** node *S_i_* is the CH and *D_m_*(*S_k_*) < *D_m_*(*S_i_*)    Add node *S_k_* as the relay node of *S_i_*  **End If****End For**

Each node can elect to become a cluster head according to the weight compared to that of its neighbors, as in State 2 in Algorithm 1. If the node has the highest weight compared to that of its neighbors, the node will broadcast the “*JOIN*” message to its neighbors in order to form a cluster, as indicated in lines 14 to 17. Otherwise, the node will wait for the “*JOIN*” message; if the node receives only one message, it will become a cluster member of the cluster. If the node receives many “*JOIN*” messages, it will select the sender node with the highest received power or the node with the nearest distance.

We assume that the cluster can be formed between the nearby nodes to reduce energy consumption and transmission time. However, because the nodes at depth *D*_2_ and *D*_3_ may not transmit data directly to the sink due to the limited transmission range, we assume that some nodes will become the CR to forward data to the sink.

State 3 of Algorithm 1 shows the steps in the selection of the relay node. Assumed nodes which are not the CH will become a CR or CM. From lines 24 to 26, if the node receives the “*JOIN*” message from both *D_m_* and *D_m_*_−1,_ which are broadcasted by nodes of upper and lower depths, the node elects to become a CR, which will be a forwarder in the routing path. From lines 28 to 29, the nodes only receive a “*JOIN*” message from the CHs at the same depth. The nodes send a “*RESPONSE*” message to the CH to confirm the cluster ID.

State 4 of Algorithm 1 and its multi-hop routing path creation show how the cluster at the deeper layer can forward the packet to the sink via the forwarders or the CR. In line 33, if the CH *S_i_* receives a “*RELAY*” message from *S_k_*, node *S_i_* checks the depth of *S_k_*. From lines 34 to 35, if node *S_k_* is located near the surface, then node *Si* or *D_m_*(*S_k_*) < *D_m_*(*S_i_*), and node *S_i_* adds *S_k_* as a relay node.

The cluster head schedules the transmission of all cluster members as a time division multiple access (TDMA). The data will be transmitted in the steady phase. The cluster head is selected at the beginning of each superframe in order to balance the load and energy for each node.

### 3.3. Qualitative Comparison of Clustering Protocols

In this subsection, we provide a qualitative comparison of the clustering protocols for UWSNs with different aspects. 

The clustering protocols can take into account many metrics which describe the network topology, such as distance to the sink or other nodes, number of neighbors, path loss, location of the node, and residual energy. Even though each protocol uses different metrics to select the cluster head, they mostly focus on the residual energy of the node and the distance between the node to the sink. To increase network reliability, some clustering protocols select an assisted node which can assume the role of the sink [9,12,18] or the control node [10]. The assisted node can receive data from the sensor node or control the network topology. As a result, the energy consumption of the nodes may be reduced by conserving the node’s energy. However, some clustering protocols are only presented and evaluated in two-dimensional (2D) deployment, whereas in the network scenarios of UWSNs, the deployment is three-dimensional (3D). To investigate the clustering protocol with an assisted node in 3D, our proposed EECMR protocol groups the sensor nodes with regard to the residual energy, cluster head duration, and distance to the sink. Among the clustering protocols, only EECMR considers the duration of the cluster head and cluster relay or assisted node in 3D, whereas the others do not consider the duration of the cluster head. A qualitative comparison of the clustering protocols is presented in Table 2. 

## 4. Performance Evaluation

We implemented the EECMR protocol and compared it to LEACH [5], DBR [7], and EERBLC [9] in terms of network lifetime and residual energy. DBR, LEACH, and EERBLC were selected for performance comparison since the three protocols consider the clustering and depth of the routing protocol. Network performance was compared using the MATLAB simulation tool. The simulation parameters were set up as in [10,12].

### 4.1. Simulation Environment

The network scenario consists of dynamic nodes deployed sparsely in a three-dimensional environment. The water current causes node movements, which change the topology rapidly. The sink is located at the center of the surface, as shown in Figure 1. The simulation parameters are shown in Table 3. According to the sensed data, the node can generate one or two packets for transmission. The transmission range of each node varies from 150 to 200 m [10,12]. The underwater sensor nodes are mobile, with the current at 1–3 m/s, which causes changes in the network deployment [10]. In the study, the network deployment was changed after every 100 rounds in order to evaluate the network’s mobility. The transmission range of nodes was varied in order to evaluate different network scenarios.

**Figure 3:**
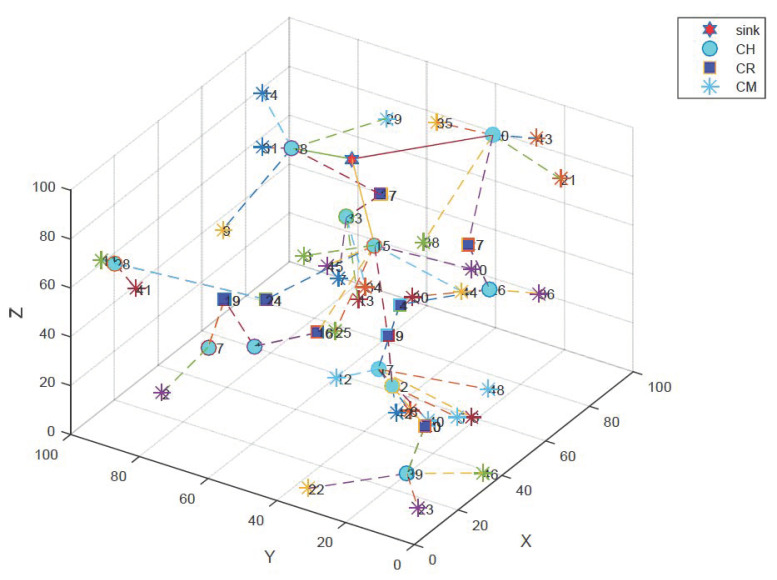
Network deployment after clustering.

An example of a network after the implementation of EECRM is shown in Figure 3. Each node is represented as a CM, CH, or CR. The sink is located at the surface, which is represented as a red star in the figure. In order to easily visualize the cluster, we deployed 50 nodes in a volume of 100 × 100 × 100 m. We assumed that the nodes nearest to the sink would become the main forwarder for other nodes, the nodes at the seabed would select the cluster to join, and some nodes at the border of two depth layers would act as the relay which connects the cluster head of the deeper cluster to the sink. 

### 4.2. Simulation Results

#### 4.2.1. Network Lifetime

**Figure 4:**
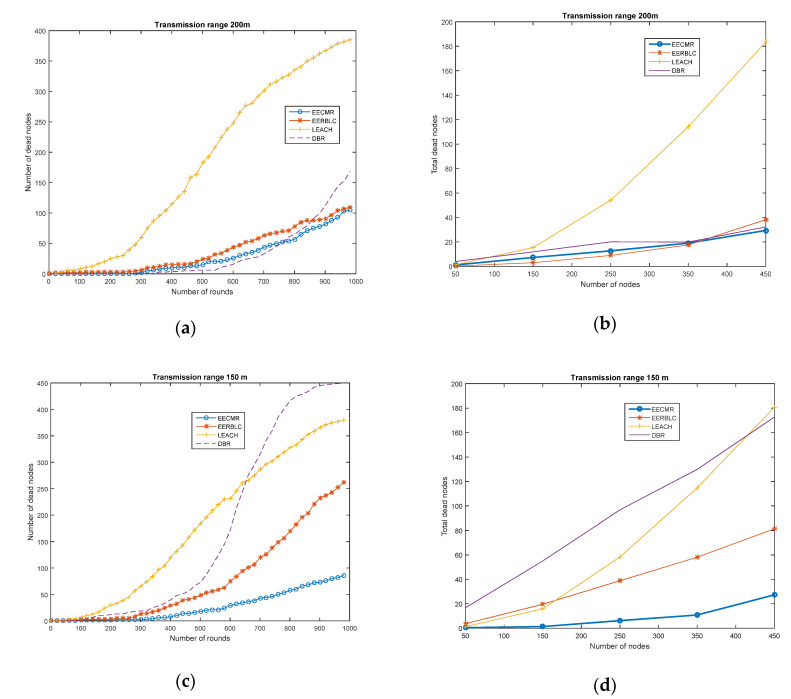
Number of dead nodes: (**a**) Transmission range 200 m, 450 nodes in 1000 rounds; (**b**) Transmission range 200 m, nodes 50 to 450; (**c**) Transmission range 150 m, 450 nodes in 1000 rounds; (**d**) Transmission range 150 m, nodes 50 to 450.

In this work, the network lifetime is defined as the total time that the nodes are alive, which can be considered the number of rounds. When the residual energy of a node decreases to zero, the node is considered a dead node. In our simulation, we compared three protocols to evaluate the network lifetime while varying the number of nodes in 1000 rounds, as shown in Figure 4. In Figure 4a, we consider a network of 450 nodes in 1000 rounds with a transmission range at 200 m; the results show that nodes began to run out of energy at round 300 for LEACH, DBR, EEBLC, and EECMR. The number of dead nodes of the proposed protocol was the lowest compared to the other protocols.

In Figure 4b, we varied the number of nodes in 1000 rounds with the transmission range at 200 m. The LEACH protocol had the highest number of dead nodes compared to the DBR, EEBLC, and EECMR. Since EECMR allows the nodes to form a cluster according to their depth level, nodes only communicate with the nodes at the same depth level in the same cluster. However, nodes in the LEACH protocol became cluster heads in turns according to the generated random number. The nodes may have consumed high energy as a result of increasing the number of transmissions during cluster formation. In EEBLC, the clustering allows unequal clusters in the networks in which nodes can elect to become cluster head considering the number of neighbors and its residual energy. As a result, the number of dead nodes in EEBLC is lower than that of DBR and LEACH, as in Figure 4. In the case of DBR, instead of forming into clusters, the nodes sent data to the sink via a multi-hop routing path according to their depth level. The node would send packets to the forwarder node at the upper depth level without considering the number of neighbors sending packets to the same destination. The forwarder node may have had a high load, which causes high energy consumption while sending packets. 

In Figure 4c,d, we evaluate the network performance when the transmission range was 150 m. It is clear that the network performance depended on the transmission range of the node. The number of dead nodes of the three protocols was higher when the transmission range decreased. However, the network performance of clustering protocols of LEACH, EEBLC, and EECMR was similar, which was better than DBR. Therefore, the clustering protocol showed better performance than the multi-path routing protocol.

#### 4.2.2. Residual Energy at the Nodes

**Figure 5:**
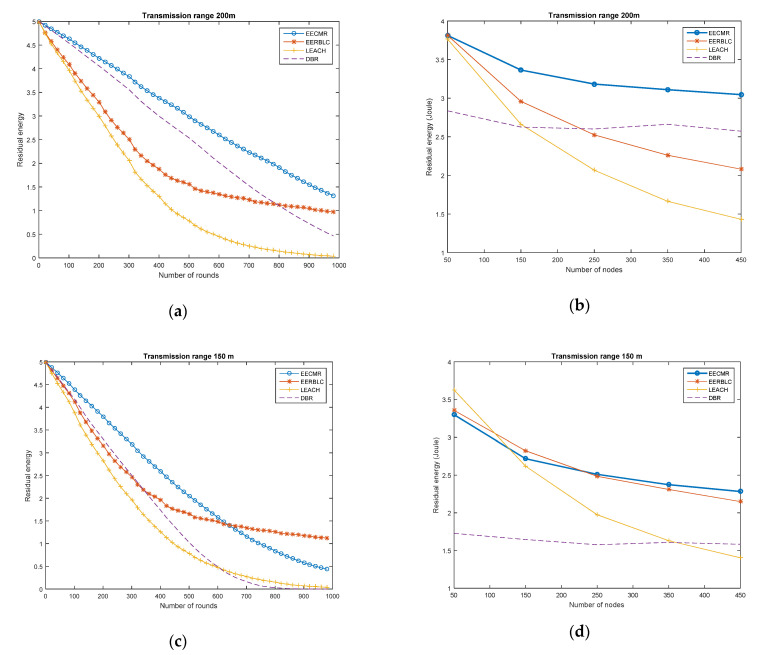
Average residual energy of a node: (**a**) Transmission range 200 m, 450 nodes in 1000 rounds; (**b**) Transmission range 200 m, nodes 50 to 450; (**c**) Transmission range 150 m, 450 nodes in 1000 rounds; (**d**) Transmission range 150 m, nodes 50 to 450.

In Figure 5, the residual energy of the nodes is shown in different network scenarios. At each round, the nodes generated packets and then forwarded them to the sink node. In Figure 5a, the residual energy of the node decreased according to the timeline, while the number of rounds increased. It is clear that our EECMR performed better in terms of conserving energy; the residual energy in EECMR was higher than that of DBR, LEACH, and EEBLC. DBR was developed for routing protocols in underwater wireless sensor networks, so DBR performed better than LEACH. EEBLC allows nodes to become the cluster head in turns according to their location and residual energy, and the nodes can save energy when varying the number of nodes and transmission range. 

In Figure 5b, we varied the number of nodes in the network, while three protocols decreased the residual energy. Despite this, the residual energy in EECMR was higher than in EEBLC, LEACH, or DBR. However, the residual energy of the nodes in LEACH declined quickly compared to EECMR. This can be explained as follows. In a dense network deployment, the nodes consume higher energy to perform more communication tasks, such as transmitting and receiving packets to the larger number of nodes. The nodes in EECMR can act as a cluster head or cluster relay; therefore, the cluster head has the information of its cluster member, which reduces the number of transmissions between nodes. As noted in the previous section, the number of dead nodes in EECMR is less because the nodes conserve energy while sending packets. EEBLC performs better than the others in the case of a low transmission range which ensures a long network lifetime. In LEACH, the node that elects to become a cluster head broadcasts information to the three-dimensional area network; as a consequence, the node consumes more energy. In DBR, the node selects the upper-level depth to become its forwarder. If the number of nodes increases, the forwarder may receive more packets, and then it sends these to the upper-level depth before reaching the sink. More transmission at the nodes and forwarders leads to high energy consumption, as shown in Figure 5. 

In Figure 5c,d, the decrease in the transmission range led to higher energy consumption in EECMR, EEBLC, LEACH, and DBR. These protocols gradually diminished the residual energy with respect to the number of rounds. When increasing the number of rounds with a low transmission range, EERBLC performs better than EECMR. When the number of nodes increases, LEACH and EEBLC perform better than EECMR in the case of a low number of nodes; EECMR performs better than LEACH in the case of dense deployment. 

In a comparison of the residual energy performance with different transmission ranges in EECMR, the residual energy for a 200 m transmission distance was higher than a 150 m transmission range. This can be explained as follows. The cluster heads in EECMR aggregate the data of all cluster members and then forward these to the cluster relay which belongs to another cluster. Since the number of data packets is re-transmitted via multi-hop, the amount of energy consumption increases. In addition, the change in the network topology causes the re-established cluster to consume energy-transmitting and -receiving control packets. However, the total received packets at the sink should be considered. This is presented in the next section.

#### 4.2.3. Received Packets at the Sink Node

**Figure 6:**
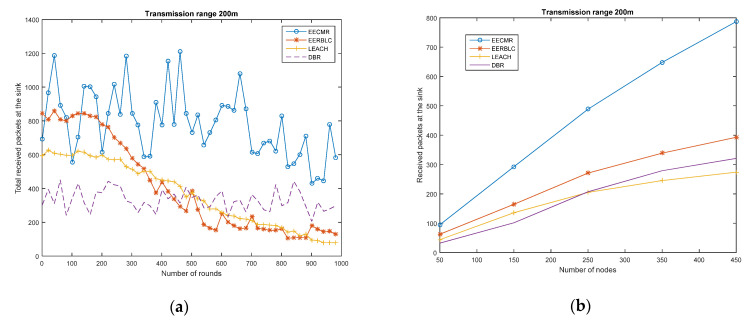

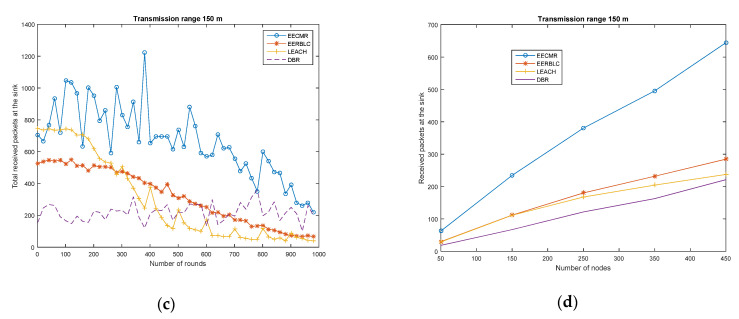
Received packets at the sink: (**a**) Transmission range 200 m, 450 nodes in 1000 rounds; (**b**) Transmission range 200 m, nodes 50 to 450; (**c**) Transmission range 150 m, 450 nodes in 1000 rounds; (**d**) Transmission range 150 m, nodes 50 to 450.

We assume that all the sensed data of nodes will be received at the sink. In the network deployment, the network topology changes every 100 rounds, which may cause a failure to receive packets at the cluster head and cluster relay. This is because the distance from a cluster member to the cluster head is greater than the transmission range and the received packets at the cluster head or cluster member may fail as a result of a low level of received power. Therefore, the cluster must be re-established, and a new cluster head, cluster member, and cluster relay must be selected. In Figure 6, the total received packets at the sink are evaluated in four cases. In Figure 6a,c, despite different transmission ranges, the received packets in EECMR are higher than in LEACH, EEBLC, or DBR. It is noted that the received packets at the sink increase when the transmission range increases. The number of total received packets in the EECMR scheme is occasionally different, which causes high jitters. Despite this, the increase in transmission range leads to a lower residual energy at the node, as shown in Figure 5. This can be considered a pay-off with respect to the transmission range and network performance.

In Figure 6b,d, the received packets at the sink are shown according to the number of rounds. Due to the number of packets at the sensor node or the number of clusters at each round, the results in the timeline fluctuate. In total, the received packets at the sinks in EECMR are higher than other protocols, which results in high throughput and a more reliable network.

## 5. Conclusions

In this work, we propose the energy protocol EECMR for routing data packets in UWSNs. EECMR is a depth-based clustering protocol that uses the depth level of the node to select cluster head nodes and forwarder nodes for multi-hop routing. EECMR considers the residual energy of the node which elects cluster heads in turns. The nodes can change roles as cluster head, cluster member, and cluster relay. The cluster relay node forwards data from a deeper level to the sink. With the aid of a cluster relay, the energy consumption for transmission is decreased, leading to fewer dead nodes. The simulation results showed that EECMR achieves better performance in terms of higher residual energy, longer network lifetime, and higher received packets at the sink. Although the proposed protocol can properly select the cluster head and cluster relay according to the depth level and residual energy, the high energy consumption at the cluster relay between different depth levels may result in re-clustering or frequent re-clustering. However, the clustering protocol may cause high latency due to the multi-hop routing path. The different types of sensor nodes will have different data priorities, an issue which must be addressed in future work. 

In addition, several issues remain open for future work, including optimization of the network topology and implementation of the clustering protocol in firmware to support UWSN application in oceans. In our future work, we will implement the clustering protocols into firmware to investigate the practical performance. 

## Figures and Tables

**Table 1:** Table of notations.

Notations	Description
*N*	Number of nodes
*S*_0_	Sink node
*S*_i_	Sensor node index *i*, 1 ≤ *i* ≤ *N*
*L* × *L* × *L*	Three-dimensional network deployment
*Tx*	Transmission range of node
*n_depth_*	Number of layers
*Nei*(*S_i_*)	List of neighbors of node *S_i_*
*D_m_*	Depth layer index *m*, 0 ≤ *m* ≤ *n_depth_*
*Lst*(*D_m_*)	List of sensor nodes at depth *D_m_*
*TL*	Transmission loss of acoustic signal
α*_TL_*(*f*)	Absorption coefficient for the medium
*W_i_*	Weight of sensor node *S_i_*
*d*(*i*,*j*)	Distance between node *S_i_* and node *S_j_*
*d*(*i*,*S*_0_)	Distance between node *S_i_* and the sink

**Table 2:** Qualitative comparison of clustering protocols.

Protocols	Parameters to Select the Cluster Head	Network	Assisted Node	Energy
LEACH [5]	Random value	2D	None	Yes
DBR [7]	Depth-based, holding time of packets	3D	None	Yes
EERBLC [9]	Residual energy, random value, number of layers	3D	Two sinks	Yes
C-LEACH [10]	Distance, location of the node	2D	Control node	Yes
MLCEE [12]	Fitness value, small Hop_ID and low number of layers	3D	Two sinks	Yes
TCEB [13]	Residual energy, number of neighbors, path loss	2D	None	Yes
EOCA [14]	Number of neighbor nodes, residual energy, distance to the sink node	2D	None	Yes
EULC [15]	Residual energy, distance to the sink node	3D	None	Yes
S-BEAR [16]	Distance to the sink	2D	None	Yes
CVBF [18]	Residual energy, vector-based routing	3D	Virtual sink	Yes
EECMR	Residual energy, duration of the cluster head, distance to the sink	3D	Cluster relay	Yes

**Table 3:** Simulation parameters.

Simulation Parameters	Value
Network deployment area	500 × 500 × 500 m
Number of nodes	50 to 450
Generated packets at each node	1 or 2 data packets
Transmission range	150 m, 200 m
Acoustic frequency	30 kHz
Transmit power	2 W
Receive power	0.1 W
Initial node energy	5 Joules
Number of sink nodes	1
Data packet size	200 bits
Rounds	1000

## Data Availability

Data is contained within the article.

## References

[1] Felemban E., Shaikh F.K., Qureshi U.M., Sheikh A.A., Qaisar S.B. (2015). Underwater sensor network applications: A comprehensive survey. Int. J. Distrib. Sens. Netw..

[2] Awan K.M., Shah P.A., Iqbal K., Gillani S., Ahmad W., Nam Y. (2019). Underwater wireless sensor networks: A review of recent issues and challenges. Wirel. Commun. Mob. Comput..

[3] Jouhari M., Ibrahimi K., Tembine H., Ben-Othman J. (2019). Underwater wireless sensor networks: A survey on enabling technologies, localization protocols, and internet of underwater things. IEEE Access.

[4] Xu G., Shen W., Wang X. (2014). Applications of wireless sensor networks in marine environment monitoring: A survey. Sensors.

[5] Akyildiz I.F., Pompili D., Melodia T. (2005). Underwater acoustic sensor networks: Research challenges. Ad Hoc Netw..

[6] Heinzelman W.R., Chandrakasan A., Balakrishnan H. Energy-efficient communication protocol for wireless microsensor networks. Proceedings of the 33rd Annual Hawaii International Conference on System Sciences.

[7] Sandeep D.N., Kumar V. (2017). Review on clustering, coverage and connectivity in underwater wireless sensor networks: A communication techniques perspective. IEEE Access.

[8] Yan H., Shi Z.J., Cui J.H. (2008). DBR: Depth-based routing for underwater sensor networks. Proceedings of the International Conference on Research in Networking.

[9] Khan A., Ali I., Ghani A., Khan N., Alsaqer M., Rahman A.U., Mahmood H. (2018). Routing protocols for underwater wireless sensor networks: Taxonomy, research challenges, routing strategies and future directions. Sensors.

[10] Zhu F., Wei J. (2018). An energy efficient routing protocol based on layers and unequal clusters in underwater wireless sensor networks. J. Sens..

[11] Li Y., Wang Y., Ju Y., He R. Energy efficient cluster formulation protocols in clustered underwater acoustic sensor networks. Proceedings of the 2014 IEEE 7th International Conference on Biomedical Engineering and Informatics.

[12] Durrani M.Y., Tariq R., Aadil F., Maqsood M., Nam Y., Muhammad K. (2019). Adaptive node clustering technique for smart ocean under water sensor network (SOSNET). Sensors.

[13] Khan W., Wang H., Anwar M.S., Ayaz M., Ahmad S., Ullah I. (2019). A Multi-Layer Cluster Based Energy Efficient Routing Scheme for UWSNs. IEEE Access.

[14] Hong Z., Pan X., Chen P., Su X., Wang N., Lu W. (2018). A topology control with energy balance in underwater wireless sensor networks for IoT-based application. Sensors.

[15] Yu W., Chen Y., Wan L., Zhang X., Zhu P., Xu X. (2020). An Energy Optimization Clustering Scheme for Multi-Hop Underwater Acoustic Cooperative Sensor Networks. IEEE Access.

[16] Hou R., He L., Hu S., Luo J. (2018). Energy-balanced unequal layering clustering in underwater acoustic sensor networks. IEEE Access.

[17] Wang M., Chen Y., Sun X., Xiao F., Xu X. (2020). Node Energy Consumption Balanced Multi-Hop Transmission for Underwater Acoustic Sensor Networks Based on Clustering Algorithm. IEEE Access.

[18] Khan M.T.R., Ahmed S.H., Kim D. (2019). AUV-Aided Energy-Efficient Clustering in the Internet of Underwater Things. IEEE Trans. Green Commun. Netw..

[19] Ibrahim D.M., Eltobely T.E., Fahmy M.M., Sallam E.A. Enhancing the vector-based forwarding routing protocol for underwater wireless sensor networks: A clustering approach. Proceedings of the International Conference on Wireless and Mobile Communications.

[20] Gopi S., Govindan K., Chander D., Desai U.B., Merchant S.N. (2010). E-PULRP: Energy optimized path unaware layered routing protocol for underwater sensor networks. IEEE Trans. Wirel. Commun..

[21] Khasawneh A., Latiff M.S.B.A., Kaiwartya O., Chizari H. (2017). Next forwarding node selection in underwater wireless sensor networks (UWSNs): Techniques and challenges. Information.

[22] Domingo M.C. (2008). Overview of channel models for underwater wireless communication networks. Phys. Commun..

